# Myocyte enhancer factor 2C function in skeletal muscle is required for normal growth and glucose metabolism in mice

**DOI:** 10.1186/s13395-015-0031-0

**Published:** 2015-02-27

**Authors:** Courtney M Anderson, Jianxin Hu, Ralston M Barnes, Analeah B Heidt, Ivo Cornelissen, Brian L Black

**Affiliations:** Cardiovascular Research Institute, University of California San Francisco, 555 Mission Bay Blvd, South, MC 3120, San Francisco, CA 94158-2517 USA; Department of Biochemistry and Biophysics, University of California San Francisco, 555 Mission Bay Blvd, South, MC 3120, San Francisco, CA 94158-2517 USA

**Keywords:** MEF2C, Skeletal muscle, Knockout mouse, Glycogen, Glucose metabolism, Fiber type, Body size

## Abstract

**Background:**

Skeletal muscle is the most abundant tissue in the body and is a major source of total energy expenditure in mammals. Skeletal muscle consists of fast and slow fiber types, which differ in their energy usage, contractile speed, and force generation. Although skeletal muscle plays a major role in whole body metabolism, the transcription factors controlling metabolic function in muscle remain incompletely understood. Members of the myocyte enhancer factor 2 (MEF2) family of transcription factors play crucial roles in skeletal muscle development and function. MEF2C is expressed in skeletal muscle during development and postnatally and is known to play roles in sarcomeric gene expression, fiber type control, and regulation of metabolic genes.

**Methods:**

We generated mice lacking *Mef2c* exclusively in skeletal muscle using a conditional knockout approach and conducted a detailed phenotypic analysis.

**Results:**

Mice lacking *Mef2c* in skeletal muscle on an outbred background are viable and grow to adulthood, but they are significantly smaller in overall body size compared to control mice and have significantly fewer slow fibers. When exercised in a voluntary wheel running assay, *Mef2c* skeletal muscle knockout mice aberrantly accumulate glycogen in their muscle, suggesting an impairment in normal glucose homeostasis. Consistent with this notion, *Mef2c* skeletal muscle knockout mice exhibit accelerated blood glucose clearance compared to control mice.

**Conclusions:**

These findings demonstrate that MEF2C function in skeletal muscle is important for metabolic homeostasis and control of overall body size.

## Background

Skeletal muscle comprises approximately 40% of total body mass and accounts for more than 30% of the energy expenditure in the mouse. As a result, skeletal muscle plays an important role in whole-body energy homeostasis [1,2]. During exercise and insulin stimulation, skeletal muscle is the primary tissue for glucose uptake, disposal, and storage as glycogen for energy reserves [1,3]. In turn, muscle can use glycogen to produce energy through glycogenolysis, the breakdown of glycogen into glucose [4]. Increasing evidence implicates skeletal muscle as a major contributor to the development of insulin resistance since muscle is the most abundant insulin-sensitive tissue in the body. Insulin resistance leads to decreased insulin signaling and, in turn, to decreased glucose transport into muscle [5].

Sarcomeres are the functional units of muscle fibers. Sarcomeres are composed of repeating units of thick myosin filaments and thin actin filaments [6]. Depending on the type of myosin in the muscle fiber, contraction can be slow or fast [7]. Despite having a stereotyped fiber type pattern established during development, adult myofibers can switch their fiber type in response to cues such as exercise, contraction, or motor neuron activity [7-9]. Type I (slow) fibers utilize oxidative metabolism as their primary energy source, are rich in mitochondria, and are slow to fatigue. In contrast, type II (fast) fibers utilize glycolysis for energy, are fast contracting, and fatigue easily [7,10]. The proportion of fast and slow fiber types play a pivotal role in whole-body metabolism: a predominance of slow fibers leads to resistance to diet-induced obesity and an increase in fast fibers in obese mice leads to reduced body weight and fat mass [11,12].

Myocyte enhancer factor 2 (MEF2) proteins function as key transcriptional regulators of skeletal muscle development, sarcomeric gene expression, fiber type control, and glucose uptake metabolism [13-17]. MEF2 proteins undergo extensive posttranslational modifications and cofactor interactions, allowing them to function as either activators or repressors of transcription [13]. *Mef2c* is the earliest *Mef2* gene expressed in skeletal muscle [18]. Mice that lack *Mef2c* die by E10 due to cardiovascular defects [19,20], precluding studies on the role of MEF2C in skeletal muscle in germline knockout mice. Conditional inactivation of *Mef2c* in skeletal muscle on an inbred C57BL/6 background has been reported to cause defects in sarcomere integrity and postnatal muscle maturation [21]. Consistent with these observations, the *Mef2c* paralogs in zebrafish, *mef2ca* and *mef2cb,* function in sarcomere formation [22].

In this study, we used a conditional knockout approach in mice to delete MEF2C function exclusively in skeletal muscle on an outbred background. Consistent with prior studies [21,23], we found that MEF2C is required for normal fiber type composition. On the other hand, in contrast to previous studies [21], we found no evidence of lethality, and *Mef2c* skeletal muscle knockout mice survived normally. We found that mice lacking *Mef2c* in skeletal muscle have impaired overall body growth and abnormal glucose uptake and metabolism, and we found that mice lacking MEF2C in skeletal muscle display abnormal glycogen accumulation in muscle in response to exercise. Overall, our findings highlight a novel metabolic function for MEF2C in skeletal muscle, where it is required for glucose metabolism, glycogen utilization, and energy homeostasis.

## Methods

### Mice

*Mef2c*^*+/−*^, *Mef2c*^*flox/flox*^, and *Mef2c-*73k-Cre mice have been described [20,24,25]. All mice from litters intended to generate *Mef2c* skeletal muscle knockout mice (*Mef2c*^SkMKO^) were weighed on the evening of the day of birth and this was designated as P1. Overall body weight was also measured on P4, P7, P10, P14, P21, P28, and P52. Tibia length was measured at 52 days of age by dissecting the tibia and measuring the length using a vernier caliper. Voluntary exercise assays were conducted as described previously [26]. For glucose tolerance tests, 10-week-old male mice were fasted for 16 h overnight with *ad lib* access to water. Blood glucose measurements were then taken for a baseline reading (0 min) by nicking the tail with a razor blade and using a blood glucose meter (FreeStyle, Therasense, 99073-0110-01, Abbott Diabetes Care, Alameda, USA). Mice were then administered a bolus injection of glucose (1 g glucose/kg body weight). Blood glucose readings were then taken at 15, 30, 60, and 120 min. Euthanasia was performed by carbon dioxide asphyxiation followed by cervical dislocation. Genotypes were determined by Southern blot using genomic tail or embryonic yolk sac DNA. All experiments using animals complied with federal and institutional guidelines and were reviewed and approved by the UCSF Institutional Animal Care and Use Committee.

### *In situ* hybridization, histology, and immunohistochemistry

Whole-mount *in situ* hybridization was performed as previously described [27]. *Mef2c* antisense RNA *in situ* probe was generated from the plasmid pBS-MEF2C. In brief, a 208 bp fragment from the *Mef2c* cDNA was cloned into plasmid pBluescript-SKII(+) and an antisense probe was generated by linearizing the plasmid with HindIII and transcribing with T3 polymerase.

For skeletal muscle histology, muscles were isolated from mice and immediately embedded in OCT embedding medium (Tissue Tek, Thermo Fisher Scientific, Waltham, USA) and frozen in liquid nitrogen-cooled isopentane. Fresh frozen embedded muscles were then cryosectioned at a thickness of 10 μm and stained with hematoxylin and eosin (H & E) as previously described [28]. For immunohistochemistry, sections were fixed in 4% paraformaldehyde for 10 min and blocked in 3% normal goat serum in PBS for 1 h at room temperature. Sections were then incubated with mouse monoclonal anti-skeletal muscle myosin (MY-32, Sigma M4276, Sigma, St. Louis, USA) followed by incubation with Alexa Fluor 594 anti-mouse (Molecular Probes, Invitrogen, Life Technologies, Grand Island, USA). Both antibodies were used at a concentration of 1:300 in 3% normal goat serum and incubated for 1 h at room temperature. Sections were mounted using Slow-Fade Gold antifade reagent with DAPI (Invitrogen) and photographed on a fluorescence microscope. Fiber type measurement was determined using sections stained for MY32. All fibers in a 10× field from two different sections from throughout the length of the soleus muscle were counted to determine the number of Type I and Type II fibers. Tissues were prepared for electron microscopy as described previously with only minor modifications [29]. For toluidine blue staining, 1 μm sections were cut and stained with 1% toluidine blue (Sigma 89640) in 1% sodium borate.

### Periodic acid Schiff’s staining and glycogen content quantification

Periodic acid Schiff’s (PAS) staining for glycogen was done on fresh frozen 10-μm-thick sections from quadriceps muscle as previously described [30]. In brief, sections were fixed in Carnoy’s fixative (60% ethanol, 30% chloroform, 10% acetic acid) for 5 min, followed by rinsing three times with water. Sections were immersed in 0.5% periodic acid (Sigma P7875) for 5 min, followed by rinsing with water four times and then transferred to Schiff’s solution (Sigma 3952016) for 10 min, followed by rinsing in running tap water for 10 min. Sections were mounted using Cytoseal 60 mounting medium (Richard-Allan Scientific 8310-16).

Total glycogen content in muscle was quantified using an adaptation of a previously described method [31]. In brief, 30 mg of frozen gastrocnemius muscle was dissolved in 1 ml 5 N KOH in a boiling water bath, then 0.2 ml of saturated sodium sulfate and 1.5 ml ethanol were added. Samples were spun at 2,000 × *g* for 10 min at 4°C and the pelleted material was resuspended in 0.5 ml 2 N HCl and incubated in a boiling water bath for 2 to 2.5 h. Samples were cooled and neutralized to pH 6 to 8 with 4 N KOH in 0.1 M triethanolamine. Glycogen content was determined by measuring the glucose released from glycogen using a glucose assay kit as recommended by the manufacturer (Sigma GAHK20).

### RNA isolation, cDNA synthesis, and quantitative and semi-quantitative RT-PCR

RNA was isolated from the quadriceps muscle using the TRIzol protocol and purified using the RNeasy Mini Kit and RNA clean-up protocol (Qiagen, Valencia, USA), according to the manufacturer’s recommendations. To synthesize cDNA, 2 μg of total RNA was used per cDNA reaction using the Omniscript Reverse Transcriptase protocol (Qiagen). Quantitative real-time RT-PCR (qPCR) was performed on an ABI 7500 Real-Time PCR machine using SYBR Green PCR Master Mix (Applied Biosystems, Foster City, USA). The following primers were used in both the semi-quantitative and qPCR: *Mef2c*F, 5′-GGCCATGGTACACCGAGTACAACGAGC-3′; *Mef2c*R, 5′-GGGGATCCCTGTGTTACCTGCACTTGG-3′; product size: 387 bp [32]. *L7*F, 5′-GGAGCTCATCTATGAGAAGGC-3′; *L7*R, 5′-AAGACGAAGGAGCTGCAGAAC-3′; product size: 202 bp [33].

## Results

### Generation of skeletal muscle-specific *Mef2c* conditional knockout mice

**Figure 1:**
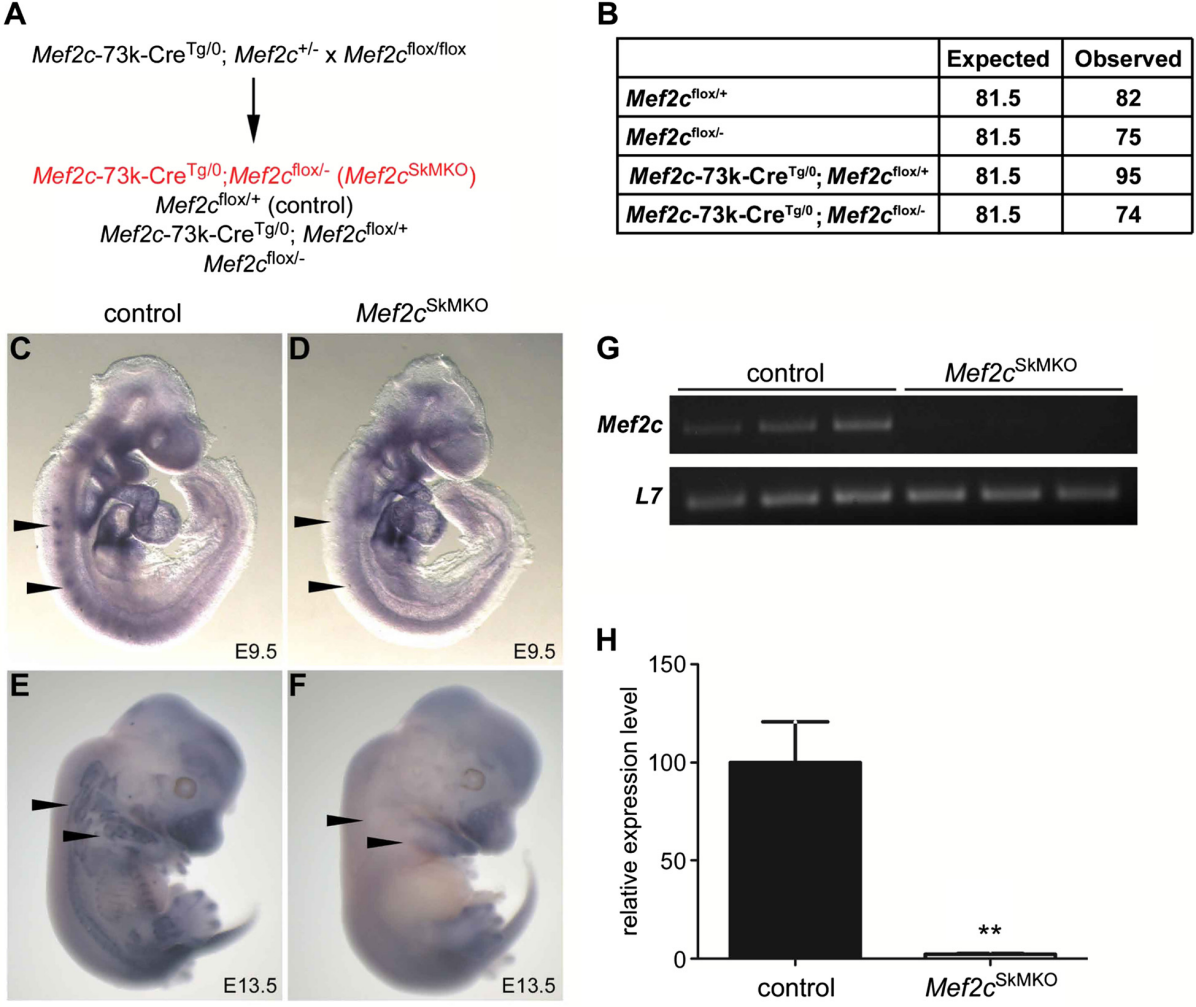
**Generation of skeletal muscle-specific** ***Mef2c*** **knockout mice. (A)** Schematic depicting the strategy for deleting *Mef2c* from skeletal muscle. *Mef2c*-73k-Cre^Tg/0^; *Mef2c* ^+/−^ males were crossed to *Mef2c* ^flox/flox^ female mice to generate control (*Mef2c* ^flox/+^) and *Mef2c* skeletal muscle knockout mice (*Mef2c* ^SkMKO^), which have the genotype *Mef2c*-73k-Cre^Tg/0^; *Mef2c* ^flox/−^ (shown in red text). **(B)** All genotypes were observed at expected Mendelian ratios (*χ* ^2^ = 0.328, 3 d.f.). (C-F) *Mef2c* was readily detected in somites of control mice at E9.5 **(C)** and in skeletal muscles of control mice at E13.5 **(E)**. In contrast, *Mef2c* transcripts were not detected by *in situ* hybridization in somites at E9.5 **(D)** or in skeletal muscles at E13.5 **(F)** in *Mef2c* ^SkMKO^ mice. **(G)** Semi-quantitative RT-PCR analyses of three control and three *Mef2c* ^SkMKO^ adult quadriceps muscle detected *Mef2c* transcripts in control but not in *Mef2c* ^SkMKO^. *L7* transcripts were similarly detected in samples from all six mice. **(H)** Expression of *Mef2c* in adult quadriceps muscle was analyzed by quantitative real-time RT-PCR (qPCR). *Mef2c* ^SkMKO^ mice showed more than 95% reduction in *Mef2c* transcripts compared to control muscle (*n* = 3; **p < 0.01). Values shown are the mean + standard deviation.

We inactivated *Mef2c* exclusively in skeletal muscle using a conditional gene targeting approach by crossing a skeletal muscle-specific Cre transgenic mouse line, *Mef2c*-73k-Cre [24], to a mouse harboring a floxed *Mef2c* allele [25] according to the strategy outlined in Figure 1A. Skeletal muscle-specific *Mef2c* knockout mice (*Mef2c*^SkMKO^) were born at predicted Mendelian frequency (Figure 1B) and were viable and fertile and appeared overtly normal (data not shown). To determine the efficiency of *Mef2c* gene inactivation in skeletal muscle, we examined the expression of *Mef2c* within the skeletal muscle in *Mef2c*^flox/+^ mice, hereafter referred to as control (ctl) mice, and *Mef2c*^SkMKO^ mice (cko) by *in situ* hybridization (Figure 1C,D,E,F). At E9.5, control embryos showed strong expression of *Mef2c* in the myotomal compartment of the somites (Figure 1C). *Mef2c* expression in the skeletal muscle of the limbs and in intercostal muscles was also readily apparent in control embryos at E13.5 (Figure 1E). By contrast, *Mef2c* expression was disrupted in *Mef2c*^SkMKO^ embryos such that it was not detectable by *in situ* hybridization at any stage examined, including E9.5 and E13.5 (Figure 1D,F). *Mef2c* transcripts were not detectable in the quadriceps muscle in adult *Mef2c*^SkMKO^ mice by RT-PCR under conditions where *Mef2c* expression was readily detectable in control mice (Figure 1G). When quantified by qPCR, *Mef2c* transcripts were approximately 50-fold more abundant in quadriceps muscle tissue from control mice than from *Mef2c*^SkMKO^ mice (Figure 1H). These results indicate a near complete inactivation of *Mef2c* in skeletal muscle using our conditional approach.

### MEF2C is required in skeletal muscle for normal growth of mice

**Figure 2:**
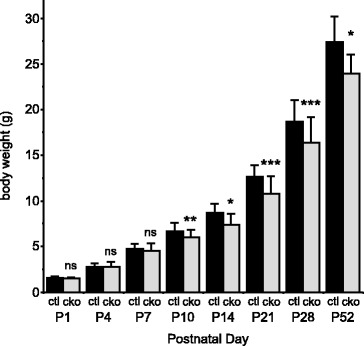
***Mef2c*** **function in skeletal muscle is required for normal growth of mice.** Body weight measurements of male mice at postnatal days (P) 1, 4, 7, 10, 14, 21, 28, and 52 (*n* = 7 to 46) show a significant difference in body weight between control (ctl) and *Mef2c* ^SkMKO^ (cko) by P10. *p < 0.05; **p < 0.01; ***p < 0.001. Values shown are mean + standard deviation.

Mice lacking *Mef2c* function in skeletal muscle appeared overtly normal and showed no decrease in viability compared to littermate controls. Interestingly, however, *Mef2c*^SkMKO^ mice weighed significantly less than age- and sex-matched controls. In male mice, this difference became apparent by postnatal day 10 (P10) and continued until early adulthood (Figure 2). The difference in weight between control and *Mef2c*^SkMKO^ mice reflected a change in overall body size, which was also apparent when the length of the tibia was measured in control and *Mef2c*^SkMKO^ mice. The tibia length in *Mef2c*^SkMKO^ mice was significantly smaller than age- and sex-matched control mice at 52 days of age (control mice, 176.14 mm ± 2.36 mm, *n* = 7; *Mef2c*^SkMKO^ mice, 168.92 mm ± 1.95 mm, *n* = 12; *p* = 0.034). Taken together, these data show that overall body size and growth is reduced when *Mef2c* is absent from skeletal muscle.

### MEF2C is required for normal fiber type composition in skeletal muscle

**Figure 3:**
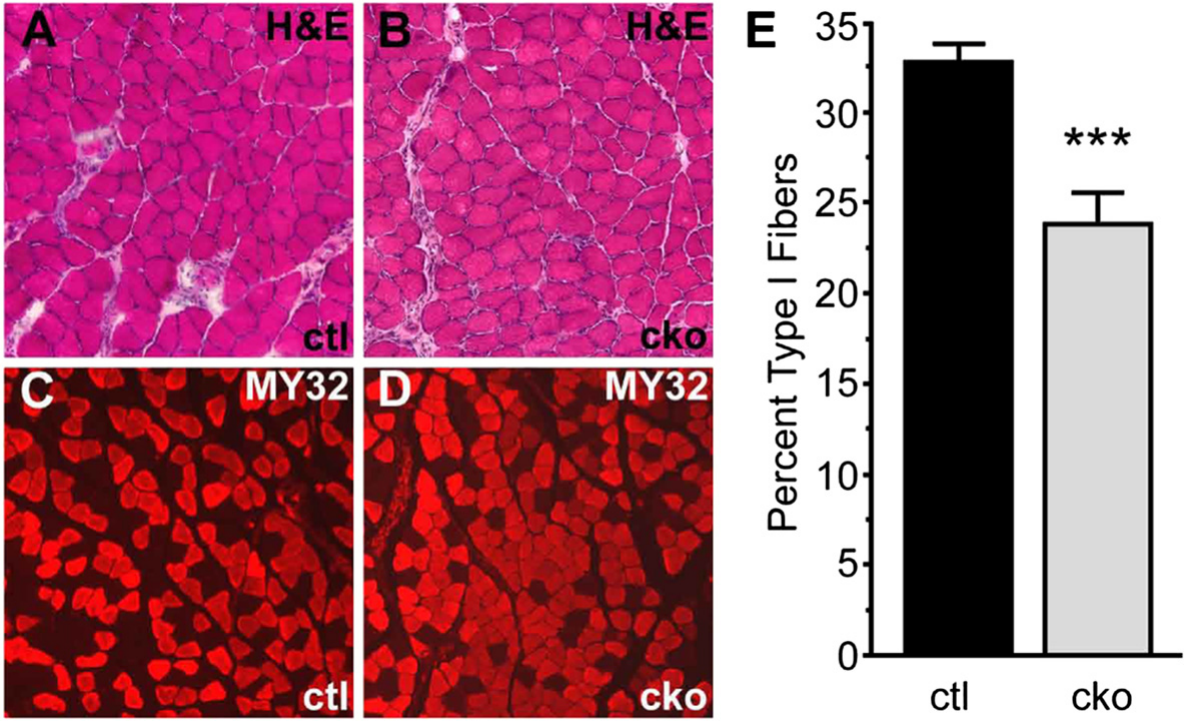
***Mef2c*** **is required for normal fiber type composition in skeletal muscle.** Soleus muscle sections from control (ctl) and *Mef2c* ^SkMKO^ (cko) male mice at 52 days of age were analyzed for morphology and fiber type. **(A, B)** Hematoxylin and eosin stain for morphological analysis on control and *Mef2c* ^SkMKO^ muscle sections at 10× magnification showed no overt differences in skeletal muscle morphology. **(C, D)** Immunofluorescence staining for fast myosin (MY32) for fiber type identification from control and *Mef2c* ^SkMKO^ muscle sections at 10× magnification showed a greater percentage of fast fibers in *Mef2c* ^SkMKO^ muscles than in controls. Red staining marks fast/type II fibers; slow/type I fibers are unstained. **(E)** Quantification of slow/type I fibers as detected by MY32 immunofluorescence staining in control and *Mef2c* ^SkMKO^ muscle sections showed a significant decrease in the number of slow fibers in *Mef2c* ^SkMKO^ mice (25.4% ± 1.2% slow) compared to control mice (32.8% ± 0.8% slow); *n* = 8; ***p < 0.001. Values shown are the mean + standard deviation.

Morphological analyses of skeletal muscle from *Mef2c*^SkMKO^ mice by H & E staining did not demonstrate any alterations in the patterning or development of skeletal muscle fibers in muscle from *Mef2c*^SkMKO^ mice compared to controls, and centrally located nuclei were not observed (Figure 3A,B). These observations suggest that muscle development, sarcomere assembly, and muscle ultrastructure were grossly normal in mice lacking *Mef2c* in skeletal muscle.

Fiber type switching from fast, glycolytic type II to slow, oxidative type I fibers is known to be regulated by MEF2 proteins [23,34,35]. Therefore, we examined the expression of fast myosin isoforms in the soleus muscle of control and *Mef2c*^SkMKO^ mice (Figure 3C,D). The soleus muscle is one of the few muscles in mice with a large proportion of slow fibers [36]. Staining sections of soleus muscle with MY32, an antibody that recognizes all fast myosin isoforms, showed a significantly higher percentage of fast fibers in *Mef2c*^SkMKO^ mice than in control mice (Figure 3C,D, red immunofluorescence). Quantification of fast and slow fibers indicated that mice lacking *Mef2c* in skeletal muscle had a significant decrease in the percentage of slow fibers in the soleus muscle (Figure 3E). These results are consistent with a role for MEF2C in maintaining fiber type balance, as has been previously reported [23,34,35].

### Excess glycogen accumulation in skeletal muscle of *Mef2c*^SkMKO^ mice in response to exercise

**Figure 4:**
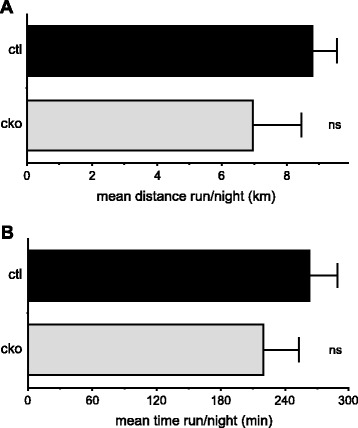
***Mef2c*** **function in skeletal muscle is not required for voluntary exercise capability.** Six-week-old male control (ctl) and *Mef2c* ^SkMKO^ (cko) mice were tested for their ability to run on an exercise running wheel for 1 week, following a 3-day training period, and distance **(A)** and time **(B)** run nightly were measured. No significant differences in either parameter were recorded; *n* = 8 to 12. Values shown are mean + standard deviation.

Because *Mef2c*^SkMKO^ mice have a reduced number of type I (slow) fibers, we examined skeletal muscle function by assessing the running ability of these mice in a voluntary exercise assay (Figure 4). *Mef2c*^SkMKO^ mice showed no significant change in the distance run or time spent running when compared to control mice despite a reduction in slow fibers (Figure 4A,B).

**Figure 5:**
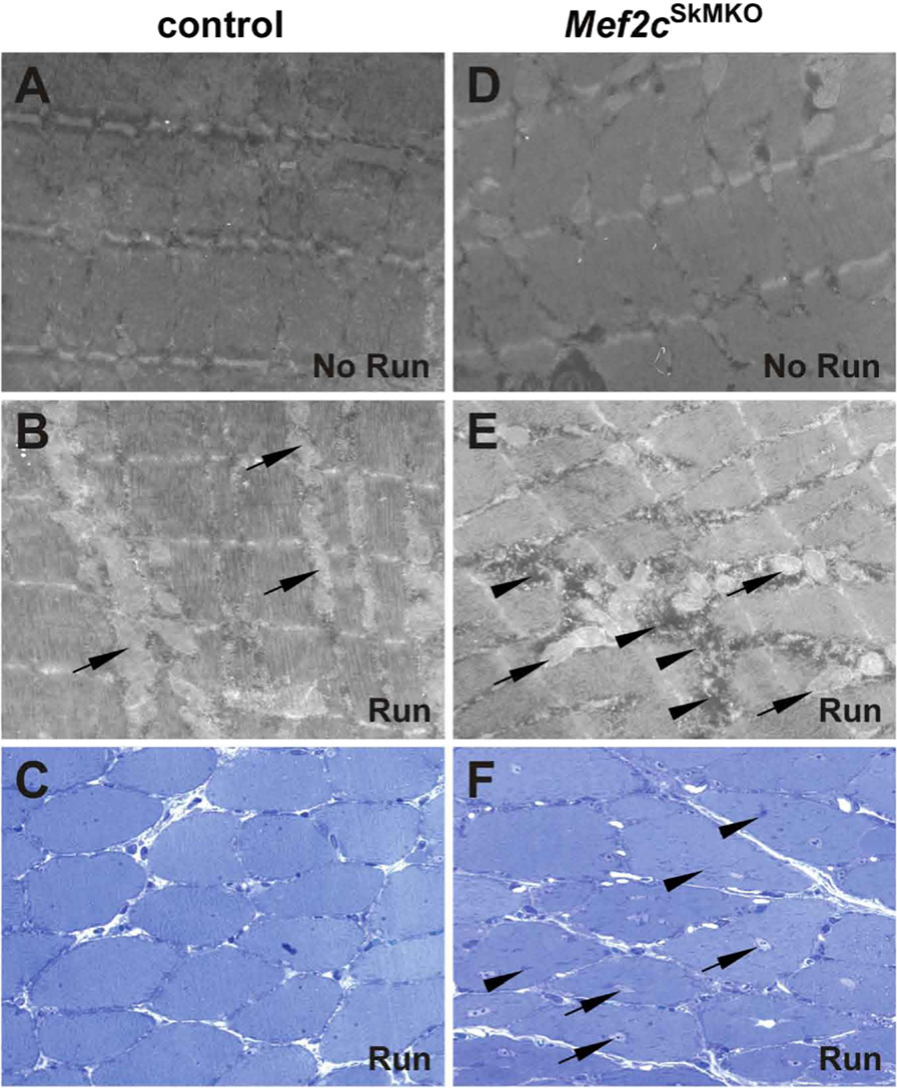
**Loss of** ***Mef2c*** **function in skeletal muscle results in exercise-induced inclusions in muscle. (A, B, D, E)** Ultrastructural analysis of the soleus muscle from exercised (Run) and unexercised (No Run) mice was determined by electron microscopy (EM) at 8,000× magnification. **(A, D)** Unexercised control **(A)** and *Mef2c* ^SkMKO^ **(D)** mice each displayed well-organized sarcomeres prior to exercise. **(B, E)** After exercise, both control and *Mef2c* ^SkMKO^ mice had an increase in mitochondria (arrows). Exercised *Mef2c* ^SkMKO^ mice also had increased accumulation of large vacuolar inclusion bodies (arrowheads) in between the sarcomeres **(E)**. **(C, F)** Sections from exercised control and *Mef2c* ^SkMKO^ mice stained with toluidine blue and imaged at 20× magnification revealed inclusions (arrows) and centrally located nuclei (arrowheads) within the muscle fibers of *Mef2c* ^SkMKO^ mice following exercise **(F)**.

Previous studies have reported a role for MEF2C in sarcomere architecture [21]. Therefore, we examined *Mef2c*^SkMKO^ and control mice for sarcomeric structural defects at baseline and following a weeklong voluntary exercise regime by electron microscopy (Figure 5). Non-exercised (No Run) control and *Mef2c*^SkMKO^ mice had well-organized sarcomeres and essentially no difference in the ultrastructure of the muscle (Figure 5A,D). Analyses of post-exercise (Run) skeletal muscle demonstrated an increased number of mitochondria in both control and *Mef2c*^SkMKO^ mice compared to mice that had not been subjected to voluntary exercise (Figure 5B,E). This was an expected result since increased mitochondrial biogenesis is a normal response to exercise [37]. Importantly, we observed the presence of large vacuolar inclusion bodies in the soleus muscle of *Mef2c*^SkMKO^ mice following 7 days of voluntary exercise (Figure 5E, arrowheads). These vacuoles were present throughout the muscle and qualitatively appeared to contain a granular material (Figure 5E). Toluidine blue staining also suggested the presence of inclusions, as well as centrally located nuclei, in the muscle fibers of exercised *Mef2c*^SkMKO^ mice but not in control muscle (Figure 5C,F). These results support a role for MEF2C in maintaining muscle in a normal healthy state in response to a period of extended exercise.

**Figure 6:**
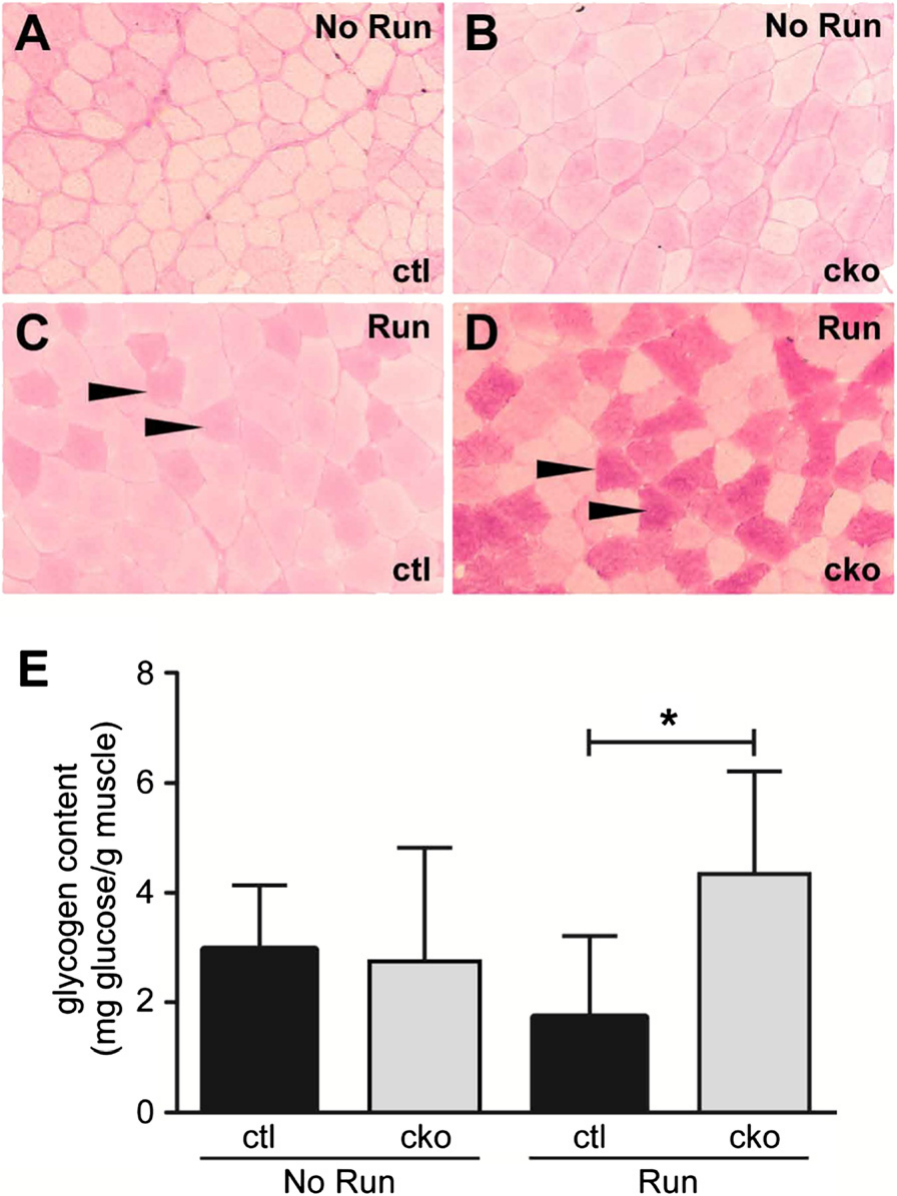
***Mef2c*** **is required for normal glycogen utilization in response to exercise.** **(A, B, C, D)** Glycogen content was qualitatively analyzed in the soleus muscle from exercised (Run) and unexercised (No Run) control (ctl) and *Mef2c* ^SkMKO^ (cko) mice by periodic acid Schiff’s (PAS) stain at 10× magnification. Arrowheads depict regions of glycogen accumulation in exercised mice **(C, D)**. Red staining marks glycogen staining. **(E)** Glycogen concentration was quantified by measuring the amount of glucose resulting from the breakdown of glycogen in the gastrocnemius muscle from unexercised (No Run) and exercised (Run) control and *Mef2c* ^SkMKO^ mice. *Mef2c* ^SkMKO^ mice abnormally accumulated glycogen in muscle in response to exercise compared to unexercised *Mef2c* ^SkMKO^ mice and compared to exercised control mice, *p < 0.05. Data are presented as milligrams of glucose per gram of muscle tissue used in the assay. Values shown are mean + standard deviation, *n* = 9.

The granular vacuoles in *Mef2c*^SkMKO^ mice superficially resembled glycogen deposits. Therefore, we examined glycogen levels in soleus muscles from control and *Mef2c*^SkMKO^ mice pre- and post-exercise (Figure 6). PAS staining for glycogen showed that control and *Mef2c*^SkMKO^ mice had similar glycogen levels under baseline (No Run) conditions (Figure 6A,B). However, control mice had a slight drop in glycogen levels post-exercise (Figure 6C). Remarkably, *Mef2c*^SkMKO^ mice actually displayed an increase in glycogen content in response to exercise (Figure 6D, dark staining). Quantification of the glycogen in the muscles of these mice, as determined by glycogen precipitation and glucose release from glycogen, confirmed a significant increase in glycogen in *Mef2c*^SkMKO^ mice in response to exercise compared to control run mice (Figure 6E). Taken together, these results demonstrate that MEF2C is necessary for appropriate glycogen metabolism in skeletal muscle in mice in response to exercise.

### MEF2C is required in skeletal muscle for normal glucose homeostasis

**Figure 7:**
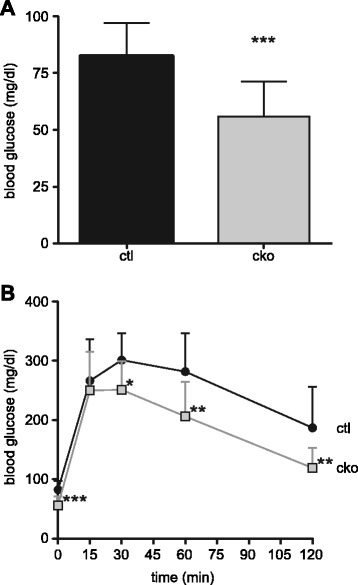
***Mef2c*** **function in skeletal muscle is required for normal glucose homeostasis.** Ten-week-old control (ctl) or *Mef2c* ^SkMKO^ (cko) male mice were tested for glucose tolerance. **(A)** Following a 16-h fast and prior to glucose injection, *Mef2c* ^SkMKO^ (cko) had significantly lower blood glucose than control (ctl) mice, *n* = 12. **(B)** After a 16-h fast, mice received a bolus injection of glucose (1 g glucose/kg body weight) at time = 0. After an initial rise in blood glucose that resulted in statistically identical blood glucose at *t* = 15 min, *Mef2c* ^SkMKO^ mice cleared glucose from the blood more quickly than control mice, *n* = 12. Blood glucose readings were taken at 0, 15, 30, 60, and 120 min post-injection. *p < 0.05; **p < 0.01; ***p < 0.001. Values are mean + standard deviation.

The abnormal synthesis or metabolism of glycogen and the overall smaller body size in *Mef2c*^SkMKO^ mice suggested that MEF2C function in skeletal muscle might be important for normal whole body glucose homeostasis. To test this hypothesis, we measured blood glucose in control and *Mef2c*^SkMKO^ mice after 16 h of fasting (Figure 7A). We observed that *Mef2c*^SkMKO^ mice had significantly lower blood glucose levels compared to control mice (Figure 7A). Furthermore, when mice were given a bolus injection of glucose after 16 h of fasting, *Mef2c*^SkMKO^ mice cleared the glucose from their bloodstream more quickly than control mice (Figure 7B). Taken together, these data suggest that MEF2C is required in skeletal muscle for normal whole body glucose metabolism and homeostasis.

## Discussion

MEF2 proteins function as key transcriptional regulators in skeletal muscle. Several metabolic genes have been identified as direct targets of MEF2 [13,16,38], including the genes encoding the glucose transporter GLUT4, the master regulator of metabolism and mitochondrial biogenesis PGC-1α, and muscle creatine kinase, which is required for the production of ATP in skeletal muscle [17,39-42]. In the present study, we found that MEF2C is required in skeletal muscle for overall body growth, which raises the interesting question as to how MEF2C function in skeletal muscle affects whole body size. Skeletal muscle is the primary tissue for insulin-stimulated glucose uptake, disposal, and storage as glycogen for energy reserves [1,17]. Intriguingly, *Mef2c*^SkMKO^ mice accumulate more glycogen in skeletal muscle after exercise compared to control mice. This result suggests that muscle requires MEF2C function to induce a switch in substrate utilization to glycogen such that in *Mef2c*^SkMKO^ mice there is an alteration of the glucose import and glycogen storage pathways. Because *Mef2c*^SkMKO^ mice have lower blood sugar levels after fasting and clear glucose from the bloodstream more quickly than control mice, our data suggest that MEF2C-dependent pathways are important for maintaining a normal balance between glucose and glycogen. The accumulation of glycogen in the skeletal muscle of *Mef2c*^SkMKO^ mice in response to exercise could be due to importing too much glucose into the muscle during exercise and then converting it to glycogen for storage [17]. Alternatively, MEF2C may be required to break down glycogen to use as an energy source. Indeed, several genes involved in glycogen metabolism, including those encoding GLUT4 and glycogen phosphorylase, have been established as direct transcriptional targets of MEF2 or implicated in MEF2-dependent gene regulation [40,43-45]. We examined the expression of several regulators of glucose and glycogen metabolism that might account for the alterations in glucose metabolism and glycogen storage observed in *Mef2c*^*SkMKO*^ mice, including genes encoding GLUT1, GLUT4, glycogen phosphorylase, glycogen synthase, phosphoinositide (PI) 3-kinase, and AMP kinase α2. Surprisingly, however, no changes were observed in the steady state mRNA level of any of the glucose or glycogen regulatory genes that we examined in skeletal muscle at baseline or in response to exercise (data not shown). Importantly, other MEF2 proteins, most notably MEF2A and MEF2D via their interactions with class II histone deacetylases and the CamKII pathway, are also associated with glucose uptake in skeletal muscle [17], so a role for MEF2C in glucose metabolism in skeletal muscle may involve complex interactions with other MEF2 proteins.

Interestingly, deletion of the bHLH transcription factor myogenin from skeletal muscle at either E15.5 or E17.5 results in smaller overall body size through an unknown mechanism [46]. Myogenin interacts with MEF2C to activate skeletal muscle differentiation [47], suggesting the possibility that MEF2C and myogenin may directly regulate genes necessary for proper glucose import and glycogen storage, such as *Glut4* or other genes associated with insulin signaling.

Previous studies of mice lacking *Mef2c* in skeletal muscle on an inbred C57BL/6 background demonstrated lethality by postnatal day 2 due to abnormally formed, disorganized sarcomeres and weakened M lines [21]. In contrast, in the studies presented here, we found that mice lacking *Mef2c* in skeletal muscle on an outbred background were viable and we observed no disorganization of sarcomeres in *Mef2c* skeletal muscle knockouts. We believe that the most likely explanation for this discrepancy is the difference in the genetic backgrounds of the mice in the two studies. In support of this idea, Potthoff *et al*. noted that inactivation of *Mef2c* in skeletal muscle on other genetic backgrounds, such as 129/SvEv, resulted in a less severe phenotype [23]. Another possible explanation for the differences in viability of *Mef2c* skeletal muscle knockout mice observed in the two studies might be subtle differences in the Cre lines used. In the studies presented here, we used a transgenic mouse line in which Cre is under the control of promoter and enhancer elements from the *Mef2c* gene [24]. Potthoff *et al*. used a very similar Cre line, where Cre is under the control of the same 1 kb enhancer element from *Mef2c* fused to a 1.5 kb promoter element from the *myogenin* gene [48]. Although both these Cre lines exhibit tight skeletal muscle specificity and appear to have highly similar patterns of activity [24,48], subtle differences in the temporal or spatial pattern of Cre expression or in its level of expression might have influenced the precise degree of *Mef2c* excision. Consistent with this possibility, Potthoff *et al*. found that inactivation of *Mef2c* with *MCK*-Cre resulted in no evidence of sarcomere disorganization, suggesting that relatively subtle differences in Cre activity or pattern may also influence the severity of the phenotype observed in *Mef2c* conditional knockout mice [21]. Importantly, however, no metabolic or growth defects were reported in previous studies of *Mef2c* loss-of-function in mice [21,23].

MEF2 proteins are important regulators of fiber type in skeletal muscle. Using a multimerized MEF2-site dependent reporter mouse line, Naya *et al.* demonstrated that MEF2 was constitutively active only in slow fibers [49]. Furthermore, dephosphorylation of MEF2 by calcineurin activates MEF2 proteins and allows them to specifically activate the slow muscle fiber genes encoding troponin I slow (TnIs) and myoglobin (Mb) [34,35,50,51]. In addition, over expression of a constitutive activator form of MEF2C results in increased formation of slow fibers [23]. Consistent with these prior observations, we also found a requirement for MEF2C in maintaining the appropriate proportion of slow fibers in the soleus.

Overall, our studies demonstrate that MEF2C is required in skeletal muscle for the normal accumulation and utilization of glycogen during exercise. This observation, combined with the requirement for MEF2C for proper glucose uptake and clearance, supports a metabolic role for MEF2C in skeletal muscle, where it is required for maintaining whole body energy homeostasis and glucose metabolism. Increasing evidence implicates skeletal muscle as a major contributor to the development of insulin resistance, as muscle is the most abundant insulin-sensitive tissue in the body [52]. Insulin resistance leads to decreased insulin signaling and, in turn, leads to decreased GLUT4 translocation and glucose transport, which limits the fuel available to muscle for contraction [17]. Impaired insulin signaling also causes abnormal accumulation of lipids in skeletal muscle and reduced mitochondrial function and reduced ATP production in insulin-resistant muscle [2,53]. Because skeletal muscle is crucial to maintaining normal insulin signaling and energy production, it is important to understand the molecular mechanisms underlying skeletal muscle metabolism. A decrease in slow fibers, increased glycogen storage, and a decrease in overall body size are all consistent with the notion that mice lacking MEF2C in skeletal muscle use energy less efficiently than wild type control mice. Given the rapidly growing impact of diabetes and obesity, defining a role for MEF2C in skeletal muscle and whole body metabolism will be of great help to further understand metabolic disorders.

## Conclusions

MEF2C is a critical regulator of nearly every aspect of skeletal muscle biology, yet its role in skeletal muscle metabolism is incompletely understood, and previous genetic studies have not identified a metabolic role for MEF2C. Using as conditional knockout approach in mice, we found that MEF2C is important for the slow fiber phenotype, consistent with earlier studies. Moreover, we show that mice lacking *Mef2c* in skeletal muscle have impaired overall body growth, display abnormal glycogen accumulation in response to exercise, and have abnormal glucose metabolism. Thus, these findings highlight a novel metabolic function for MEF2C in skeletal muscle, where it is required for glucose metabolism, glycogen utilization, and energy homeostasis.

## Abbreviations

ATP: adenosine triphosphate
bHLH: basic helix-loop-helix
GLUT4: glucose transporter 4
MEF2: myocyte enhancer factor 2
MEF2C: myocyte enhancer factor 2C
PGC1α: peroxisome proliferator-activated receptor gamma coactivator 1-alpha
